# Deafness Progressing to Cochlear Implant Eligibility Is Eight Times More Likely in the Hypoplastic Than the Degenerative Endotype of Menière's Disease

**DOI:** 10.1097/MAO.0000000000004482

**Published:** 2025-03-20

**Authors:** Catrin Brühlmann, Jennifer L. Spiegel, Agnes Mühle, Adrian Dalbert, Vincent Y. W. Lin, Trung N. Le, Thore Schade-Mann, Jessica Knoppik, Dorothe Veraguth, Christof Rö,ösli, Alexander Huber, Julia Dlugaiczyk, Steven D. Rauch, Hubert Löwenheim, Joseph M. Chen, Amy F. Juliano, Andreas H. Eckhard, David Bächinger

**Affiliations:** ∗ Department of Otorhinolaryngology, Head and Neck Surgery, University Hospital Zurich, Zurich, Switzerland; † University of Zurich, Zurich, Switzerland; ‡ Department of Otolaryngology–Head and Neck Surgery, Faculty of Medicine, University of Toronto, Toronto, Ontario, Canada; § Biological Sciences Platform, Hurvitz Brain Sciences Program, Sunnybrook Research Institute, Toronto, Ontario, Canada; ∥ Department of Otorhinolaryngology, University Hospital, LMU Munich, Munich, Germany; ¶ Department of Otolaryngology–Head & Neck Surgery, University of Tübingen Medical Center, Tübingen, Germany; ∗∗ Diagnostic and Interventional Neuroradiology, Department of Radiology, University Hospital Tübingen, Germany; †† Department of Otolaryngology–Head and Neck Surgery, Harvard Medical School, Boston, MA, USA; ‡‡ Otopathology Laboratory, Department of Otolaryngology–Head and Neck Surgery, Massachusetts Eye and Ear, Boston, MA, USA; §§ Department of Radiology, Massachusetts Eye and Ear, Harvard Medical School, Boston, MA, USA

**Keywords:** Degeneration, Endolymphatic sac, Hypoplasia, Prediction, Risk, Vestibular aqueduct

## Abstract

**Objective:**

To investigate whether one of the two recently described MD endotypes—defined by either endolymphatic sac degeneration (MD-dg patients) or hypoplasia (MD-hp patients)—is associated with an increased likelihood of undergoing CI.

**Study Design:**

Retrospective multicenter cross-sectional study.

**Setting:**

Five tertiary referral centers.

**Patients:**

CI cohort: 115 adult MD patients with a history of uni- or bilateral CI. Non-CI cohort: 72 MD patients with no CI history. All included patients matched current diagnostic criteria for definite MD.

**Intervention:**

Cochlear implantation.

**Main Outcome Measures:**

Endotype distribution (MD-dg versus MD-hp) between the CI cohort and the non-CI cohort. The endotype was determined using high-resolution CT data based on the angular trajectory of the vestibular aqueduct, following established protocols. Secondary outcomes included disease laterality, age at MD diagnosis, duration of MD, and pre-CI hearing thresholds.

**Results:**

The CI cohort included significantly more MD-hp patients than the non-CI cohort (72% versus 24%, *p* < 0.0001). The odds ratio of CI for an MD-hp patient relative to an MD-dg patient was 8.4 (95% confidence interval, 4.3–16.1). Pre-CI audiometric data showed no significant differences in hearing thresholds between endotypes, neither in the implanted nor in the non-implanted ear.

**Conclusions:**

The MD-hp endotype, frequently associated with bilateral disease and early-age disease onset, is strongly linked to a higher likelihood of CI. Endotyping of MD patients based on endolymphatic sac pathology can effectively stratify their risk of severe hearing loss, guiding personalized audiological follow-up and clinical decisions regarding potential CI.

## INTRODUCTION

Progressive sensorineural hearing loss is a defining feature of Menière's disease (MD) (1) that significantly impacts patients' quality of life (2,3). Previous studies indicate that up to 7% of MD patients progress to profound hearing loss, defined as a pure-tone average (PTA) of 70 dB hearing level (HL) or worse (4), and that 24% experience severely reduced speech discrimination scores of ≤40% (5). Cochlear implantation (CI) has proven to greatly benefit these MD patients, yielding significant improvements in speech perception and quality of life, comparable to those seen in other patient populations with profound hearing loss (6,7). However, the trajectory of hearing decline among MD patients is unpredictable, and early identification of patients meeting criteria for CI would enable more personalized and timely care strategies.

Recent studies have identified two distinct MD “endotypes,” characterized by specific endolymphatic sac (ES) pathologies (8): degeneration in the MD-dg group (approx. 75% of patients) and incomplete development in the MD-hp group (approx. 25% of patients) (8,9). These two pathologies can be reliably discerned and diagnosed in clinical MD patients using temporal bone computed tomography (CT) imaging in combination with established radiological surrogate markers, including the angular trajectory of the vestibular aqueduct (ATVA) and others (10–12). Notably, these two endotypes have been linked to different clinical characteristics, such as variations in the age of disease onset and the likelihood of progression to bilateral MD (8,13). However, whether these endotypes are associated with the likelihood of profound hearing loss and the need for CI is unexplored.

This study investigated the relationship between MD endotypes and the likelihood of undergoing CI. By analyzing multicenter cohorts of patients with definite MD, both with and without a history of CI, we aimed to determine whether one endotype is more strongly associated with a predisposition for severe to profound hearing loss, thereby increasing the likelihood of CI eligibility.

## MATERIALS AND METHODS

### Patients

The study procedures were approved by the local review boards (Zurich, no. KEK-ZH 01619/01006; Munich, no. 19-086/21-0779; Toronto, no. REB2205; Tubingen, no. 016/2014BO1/612/2024BO2; Boston, no. 2022P001326). Where required, written informed general consent was obtained from all participants in accordance with local and regional ethical requirements for human studies. The study used a multicenter cross-sectional design to compare the prevalence of MD endotypes between two cohorts: (i) MD patients who had undergone cochlear implantation (“CI cohort”) and consecutively assessed, and (ii) unselected MD patients who had not undergone CI (“non-CI cohort”).

The CI cohort was recruited by screening local electronic CI databases for adult patients who had undergone CI and had a clinical diagnosis of MD. All patients fulfilled local audiological (pure-tone and/or speech audiometry) criteria for CI eligibility, with the unifying criterion being unaidable hearing loss, defined as hearing loss that does not benefit from traditional amplification. Each diagnosis was reassessed using available medical records to ensure they met the current diagnostic criteria for definite MD (International Classification of Vestibular Disorders) (14). To avoid treating two ears from the same patient as independent data, only the first implanted ear was included in patients with bilateral CI. Patients without high-resolution CT data were excluded. The non-CI cohort consisted of a historical cohort of consecutively enrolled MD patients presenting in the Interdisciplinary Center for Vertigo, Balance, and Ocular Motor Disorders at the University Hospital Zurich (tertiary referral center) from 2010 to 2015. The inclusion criteria were available imaging data and a final diagnosis of definite MD (14). This cohort has previously been characterized (9).

The study follows the Strengthening the Reporting of Observational Studies in Epidemiology (STROBE) reporting guideline (15).

### Audiometric Evaluation

All pure-tone testing was conducted following local standard procedures. Vibrotactile or questionable vibrotactile responses were treated as no response. A four-frequency air-conducted pure-tone average (PTA_4_) was calculated from hearing thresholds at 500, 1000, 2000, and 4000 Hz. Values are given as dB HL. If patients had no response at the maximum stimulation level, a value of 120 dB was used.

Because speech perception was not within the scope of the present study and due to its international, polylinguistic nature, word recognition scores were not included in the present study.

### Angular Trajectory of the Vestibular Aqueduct Measurements for Patient Endotyping

As previously described, the ATVA was used as a radiographic surrogate marker to determine the underlying histopathological subtype (degeneration versus hypoplasia of the endolymphatic sac; i.e., endotype) of the MD patients (10). These two pathologies are associated with significantly different vestibular aqueduct (VA) bending angles (*α*_exit_) as part of the ATVA, which can be reliably determined using temporal bone high-resolution CT imaging data and custom-developed software or angle measurement tools available on radiology Picture Archiving and Communication System (PACS) (10,11). As previously determined, reference values of *α*_exit_ ≤ 120° indicate a degenerative endolymphatic sac pathology, and those of *α*_exit_ ≥ 140° indicate a hypoplastic endolymphatic sac pathology (10). The ATVA was assessed either by a neurotologist, who had been involved in developing the method of evaluating the ATVA, or by one of two experienced neuroradiologists specifically trained in assessing the ATVA.

### Statistical Analysis

All statistical tests were selected before data collection. The primary variable was the endotype distribution, which was compared between the CI cohort and the non-CI cohort using the odds ratio. The odds ratio measured the likelihood of an endotype being associated with CI eligibility by comparing the endotype distribution between the two cohorts. Secondary variables included disease laterality, age at MD diagnosis, MD duration at inclusion (with disease onset defined as the time point when the diagnostic criteria for MD were first fulfilled), and audiological pure-tone thresholds in the implanted and non-implanted ear before CI. As mentioned above, only the first ear undergoing CI was included in cases of bilateral CI to avoid repeated measures bias. Unless otherwise specified, values are reported as the mean and standard deviation (SD) or as absolute numbers and percentages. Continuous variables were analyzed using a two-tailed Student *t* test for independent samples. Fisher exact test was performed for binary variables. A *p* value of less than 0.05 was considered statistically significant. Statistical analyses were performed using Prism for Apple Macintosh, version 10.2.3 (GraphPad Software, Inc., La Jolla, CA).

## RESULTS

**FIG. 1:**
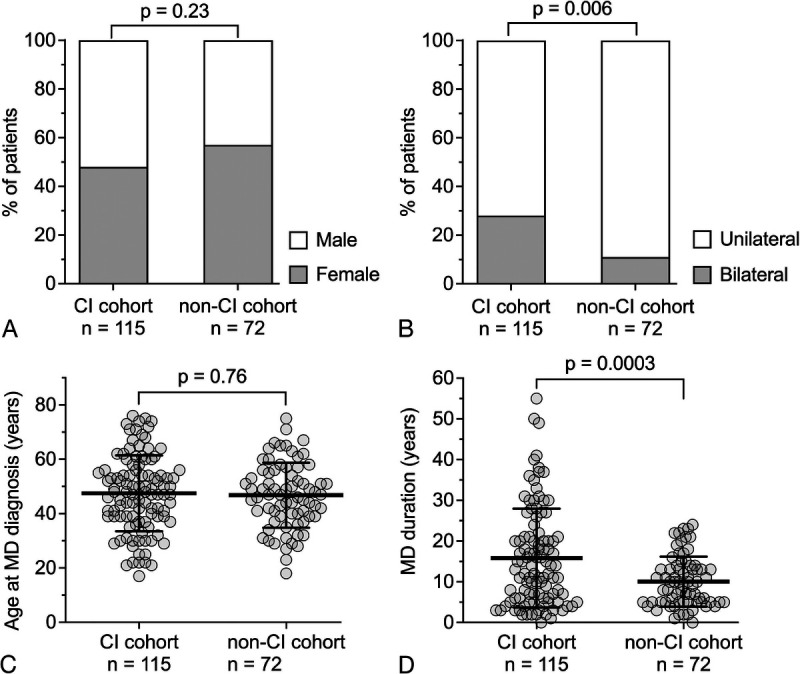
Comparison of demographic and disease characteristics between the cochlear implantation (CI) cohort and the non-CI cohort. *A*, Sex distribution. *B*, Meniere's disease (MD) laterality. *C*, Age at MD diagnosis. *D*, Duration of MD at the time of CI (CI cohort) or at the time of inclusion (non-CI cohort). In C and D, bold horizontal lines with whiskers represent the mean and standard deviation.

The initial screening identified 133 MD patients who underwent CI. In the CI cohort, a total of 115 MD patients who underwent CI at a mean age of 63.5 years (SD 11.5) between 1996 and 2023 were included. A total 18 MD patients were excluded from the CI cohort due to unavailability of imaging data, as these records were no longer accessible (e.g., deleted from archives). The non-CI cohort consisted of MD patients consecutively enrolled at a mean age of 56.8 years (SD 12.7), which has been previously characterized (9,12). There was no significant difference in the sex distribution between the cohorts (CI cohort versus non-CI cohort, female: male 48%:52% versus 57%:43%; *p* = 0.23; Fig. 1A). In the CI cohort, a significantly higher proportion of patients were bilaterally affected at the time of CI compared with the non-CI cohort at enrollment (28% versus 11%, *p* = 0.006; Fig. 1B). The age at MD diagnosis did not differ significantly between the cohorts (CI cohort versus non-CI cohort, 47.4 yr [SD 13.9] versus 46.8 yr [SD 12.0], *p* = 0.76; Fig. 1C). As expected, the duration of disease was significantly longer in the CI cohort (15.8 yr [SD 12.1] versus 10.1 yr [SD 6.1], *p* = 0.0003; Fig. 1D), reflecting the fact that profound hearing loss and CI are late-stage sequelae of MD.

**FIG. 2:**
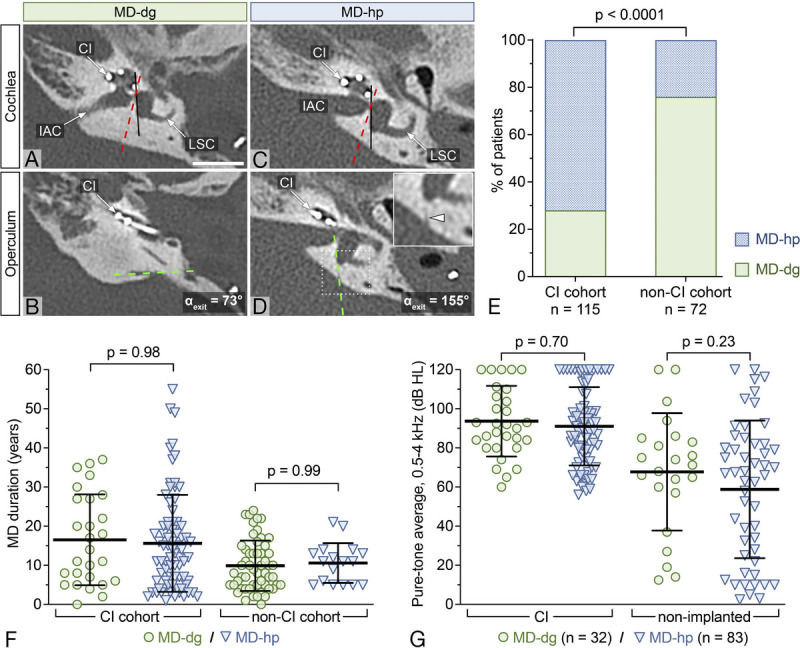
Endotyping and endotype distribution in the CI cohort. *A–D*, Representative endotyping of an MD-dg (*A–B*) and an MD-hp (*C–D*) patient from the CI cohort. The angular trajectory of the vestibular aqueduct (ATVA) is assessed in the axial plane by determining the trajectories of the proximal vestibular aqueduct (VA; red dashed line in A and C) at the level of the lateral semicircular canal (LSC) and that of the distal VA (green dashed line in B and D) at the level of the operculum. Exit angles (*α*_exit_) ≤ 120° indicate a degenerative endolymphatic sac in the MD-dg group (*A* and *B*), whereas *α*_exit_ ≥ 140° indicates a hypoplastic endolymphatic sac in the MD-hp group (*C* and *D*). Black line, medial wall of the vestibule. The inset in D highlights the barely visible operculum in a hypoplastic VA (white arrowhead). CI indicates cochlear implant electrode; IAC, internal auditory canal; scale bar, 10 mm. *E*, Endotype distribution in the CI and non-CI cohort, illustrating the proportions of MD-dg and MD-hp patients in each group. *F*, MD duration in both MD endotypes in the non-CI and CI cohort. *G*, Pure-tone average before CI in the CI cohort, grouped by endotype, for both the ear undergoing CI and the non-implanted ear.

All patients were endotyped according to previously published criteria (10), based on the morphology of the VA using the ATVA (Fig. 2A–D). In the non-CI cohort, 55 (76%) patients were classified as MD-dg and 17 (24%) as MD-hp. The CI cohort exhibited a distribution of 32 (28%) MD-dg and 83 (72%) MD-hp. Fisher exact test revealed a significantly different endotype distribution between the non-CI and CI cohort (*p* < 0.0001; Fig. 2E). The odds ratio of receiving CI for an MD-hp patient relative to an MD-dg patient was 8.4 (95% confidence interval, 4.3–16.1).

To assess disease duration as a potential confounder, we analyzed disease duration in both MD endotypes in the non-CI and CI cohort. Disease duration did not significantly differ between endotypes, either in the non-CI cohort (mean difference, 0.9 yr; *p* = 0.98) or in the CI cohort (mean difference, 0.7 yr; *p* = 0.99; Fig. 2F).

In the CI cohort, before implantation, the mean PTA_4_ was 91.5 dB (SD 20.1) in the implanted ear and 61.6 dB (SD 33.7) in the non-implanted ear. There was no significant difference in the PTA_4_ between the two MD endotypes, neither in the implanted ear (MD-dg versus MD-hp, 93.7 dB [SD 18.1] versus 91.0 dB [SD 20.0]; *p* = 0.70; Fig. 2G) nor in the non-implanted ear (MD-dg versus MD-hp, 67.8 dB [SD 30.0] versus 58.8 dB [SD 35.2]; *p* = 0.23).

## DISCUSSION

This study aimed to investigate the association between two distinct MD endotypes, characterized by different pathologies of the ES, and the likelihood of undergoing CI. Our findings demonstrate a marked predominance of MD-hp patients (72%) among CI recipients compared with the non-CI cohort (24%). Specifically, MD-hp patients were more than eight times more likely to undergo CI compared with MD-dg patients, suggesting a clear link between endotype and severe to profound hearing loss. This observed difference may even be a conservative measure given the fact that the non-CI cohort was recruited from a tertiary referral center, where a subset of individuals with more severe disease is selected.

CI is an effective treatment for severe to profound sensorineural hearing loss in MD and can also significantly mitigate tinnitus and vertigo, improving overall quality of life (6,7,16). This study, representing the largest reported cohort of MD patients undergoing CI, shows patient characteristics similar to previous studies: the PTA_4_ before implantation was 91.5 dB, consistent with previously reported 90 to 100 dB (17). The mean age at implantation was 63.5 years, aligning with the reported 62.7 to 65.4 years (7,18), although the mean duration of MD at CI was shorter (15.8 yr versus 22.8 yr) (7). Lastly, previous studies showed that 59 to 79% of MD patients undergoing CI were affected by bilateral MD (6,7). In contrast, only 28% of our MD with CI patients were bilaterally affected, which aligns well with the overall percentage of bilateral cases in the general MD population (19).

Bilateral MD has been demonstrated to occur far more often in MD-hp patients, with approximately 90% of bilateral MD patients classified as having a hypoplastic endotype (9,13). This could explain the higher proportion of MD-hp patients undergoing CI, potentially due to hearing impairment in the second ear related to bilateral MD. However, our study found no significant audiometric differences between MD-dg and MD-hp patients in the CI cohort before CI. Specifically, hearing in the non-implanted ear was comparable between MD-dg and MD-hp patients, suggesting that hearing status of the non-implanted ear does not sufficiently explain the observed endotype differences in our CI cohort. This is a surprising finding given the known higher percentage of bilateral disease in MD-hp patients (8,9,13), which deserves further investigation. Additionally, the time from disease onset to CI was similar between the two MD endotypes, indicating that hearing loss progression—and ultimately CI candidacy—is determined by the underlying endotype, rather than simply the duration of the disease. This highlights the role of histopathologic differences in determining the course of hearing deterioration.

Previous studies, however, have concluded that the audiovestibular phenotype is similar in both MD-dg and MD-hp patients (9). Therefore, different pathologies of the endolymphatic sac converge on a common final pathway, namely, a functional loss of the endolymphatic sac and decompensation of the endolymphatic system (8). This may subsequently impair the homeostatic capacity of the remaining inner ear (8). Thus, in addition to the distinct endolymphatic sac pathologies, other factors likely contribute to the hearing loss progression, particularly in the subset of MD-hp patients advancing to profound hearing loss. Notably, MD-hp patients tend to be affected by MD earlier in life (8,9), which may result in earlier deterioration of compensatory homeostatic mechanisms in the inner ear. Furthermore, MD-hp patients commonly exhibit additional developmental anomalies of the temporal bone, such as poorly pneumatized air cell tracts and a thinned, abnormally flat posterior temporal bone surface, suggesting a genetic contribution to the hypoplastic endotype of MD (12,20). This genetic factor could additionally affect the cellular and molecular architecture of the cochlea, contributing to the cochlear phenotype in MD-hp patients. Indeed, several genetic mutations and variants have been specifically associated with MD-hp patients (20), although the mechanisms by which they influence hearing remain to be investigated.

Counseling MD patients about hearing rehabilitation can be challenging due to several factors, fluctuating or bilateral hearing loss as well as progression to functional deafness, necessitating CI (7,21). Audiologic follow-up in MD has been recommended to be adapted to disease severity and progression (22). Our findings emphasize the importance of particularly close monitoring of MD-hp patients. Another challenge in MD patients is the potential development of bilateral MD, which occurs in up to 50% of patients (23). Recent studies have shown that approximately 90% of bilateral cases develop in MD-hp patients, and importantly, progression to bilateral MD can be predicted in MD-hp based on the morphology of the VA (9,13). Therefore, endotyping MD patients aids in enhancing personalized audiologic follow-up by identifying MD-hp patients who are at higher risk for both profound hearing loss and bilateral MD.

This study is limited by its retrospective design and reliance on historical data, potentially introducing selection bias and overestimating the severity and prevalence of MD-hp within the CI cohort. Furthermore, we did not report inter-rater reliability statistics for the ATVA measurements, but previous studies have indicated near-perfect agreement (11). We also assumed that patients with both MD-hp and MD-dg were equally likely to receive and accept CI recommendations. Because the study only includes patients eligible for CI, the predictive value of the MD endotype for mild and moderate hearing loss remains unknown. Further, a small subset of patients was excluded due to missing imaging data, and further analysis was not possible due to the lack of complete data as well as the small sample size, which may lead to an underpowered analysis. Moreover, the retrospective study design did not allow for sufficient control of potential confounders, such as overall health status, socioeconomic status, or geographic proximity to the treatment center. Additionally, no information on detailed vestibular function and associated counseling was included. Finally, the cross-sectional nature of the study limits the assessment of longitudinal changes in hearing and disease progression.

## CONCLUSION

MD-hp patients are more than eight times more likely to undergo CI compared with MD-dg patients. Hypoplasia of the endolymphatic sac, the underlying pathology defining this endotype, is identified as the first known critical risk factor associated with CI in the overall MD population, although the underlying pathophysiologic mechanisms remain unclear. Endotyping based on endolymphatic sac pathologies offers a novel approach to stratify MD patients by their likelihood of significant hearing deterioration. Our study highlights the clinical relevance of endotype classification in predicting disease severity and progression, which is crucial for effective counseling, audiological follow-up, and informed clinical decision-making regarding CI.
